# The values and ethical commitments of doctors engaging in macroallocation: a qualitative and evaluative analysis

**DOI:** 10.1186/s12910-018-0314-1

**Published:** 2018-07-24

**Authors:** Siun Gallagher, Miles Little, Claire Hooker

**Affiliations:** 1 Faculty of Medicine and Health, Sydney Health Ethics, Medical Foundation Building K25, University of Sydney, Sydney, NSW 2006 Australia; 2 Faculty of Medicine and Health, Health and Medical Humanities, Sydney Health Ethics, Medical Foundation Building K25, University of Sydney, Sydney, NSW 2006 Australia

**Keywords:** Macroallocation, Priority setting, Medical ethics, Values, Paul Ricoeur, Grounded moral analysis, Physicians

## Abstract

**Background:**

In most socialised health systems there are formal processes that manage resource scarcity and determine the allocation of funds to health services in accordance with their priority. In this analysis, part of a larger qualitative study examining the ethical issues entailed in doctors’ participation as technical experts in priority setting, we describe the values and ethical commitments of doctors who engage in priority setting and make an empirically derived contribution towards the identification of an ethical framework for doctors’ macroallocation work.

**Method:**

We conducted semi-structured interviews with 20 doctors, each of whom participated in macroallocation at one or more levels of the Australian health system. Our sampling, data-collection, and analysis strategies were closely modelled on grounded moral analysis, an iterative empirical bioethics methodology that employs contemporaneous interchange between the ethical and empirical to support normative claims grounded in practice.

**Results:**

The values held in common by the doctors in our sample related to the domains of personal ethics (‘taking responsibility’ and ‘persistence, patience, and loyalty to a cause’), justice (‘engaging in distributive justice’, ‘equity’, and ‘confidence in institutions’), and practices of argumentation (‘moderation’ and ‘data and evidence’). Applying the principles of grounded moral analysis, we identified that our participants’ ideas of the good in macroallocation and their normative insights into the practice were strongly aligned with the three levels of Paul Ricoeur’s ‘little ethics’: ‘aiming at the “good life” lived with and for others in just institutions’.

**Conclusions:**

Our findings suggest new ways of understanding how doctors’ values might have procedural and substantive impacts on macroallocation, and challenge the prevailing assumption that doctors in this milieu are motivated primarily by deontological considerations. Our empirical bioethics approach enabled us to identify an ethical framework for medical work in macroallocation that was grounded in the values and ethical intuitions of doctors engaged in actions of distributive justice. The concordance between Ricoeur’s ‘little ethics’ and macroallocation practitioners’ experiences, and its embrace of mutuality, suggest that it has the potential to guide practice, support ethical reflection, and harmonise deliberative practices amongst actors in macroallocation generally.

## Background

It is almost universal in contemporary western societies to construct healthcare resources as scarce and in need of rationing [1]. Most socialised health systems have formal processes in place that manage scarcity and determine the allocation of new resources to healthcare programs in accordance with their priority [2–4]. Because of its potential to impact on persons and society, health policy-making for resource allocation has been characterised as a moral endeavour [5, 6]. In this analysis, part of a larger qualitative study undertaken in NSW, Australia, we report on the values and ethical commitments of doctors who participate in health care resource allocation processes as technical experts, and make an empirically derived contribution towards the identification of an ethical framework to guide doctors who occupy this role.

Macroallocation concerns decisions that determine the amount of resources available for particular kinds of health services and programs [7–9]. Its focus on the healthcare needs of populations at an aggregate level and its locus at the level of governments and institutions distinguish it from microallocation, or bedside rationing, which concerns decisions about individual patients’ access to resources. Since it generally entails competing policy goals that require choices to be made amongst many defensible options [10] and normative assessments of the needs of groups of patients who are seen as competing for the same resources [9], it is often conceptualised as priority setting [8, 11].

In western democracies with socialised medicine, macroallocation generally makes use of the input of technical experts when deliberating options and formulating recommendations to the decision-maker, who is often a politician, but may be a local board or executive, depending on the scale of the decision and the degree to which authority is delegated in the system. In healthcare policy, doctors are the dominant technical experts [12–16]. That they are essential as expert informants seems to be readily accepted [2, 17], notwithstanding concerns about their lack of special expertise in determining the outcomes likely to promote social justice [18–20], their limitations as barometers of public preferences [21, 22], and the ubiquity of conflicts of interest and role [21, 23–26].

Values and interests underlie macroallocation deliberations [14, 25, 27] and the production and use of the medical evidence that is the focus of policy deliberation [28–30]. Usually, priority setting is guided by rules or principles that have been set by government or the relevant health authority [31]; ideally, these are consistent with societal norms and values [3]. In most systems, distributive justice is the overarching principle guiding decisions, although one or a combination of liberal, egalitarian, utilitarian or communitarian models may be used to arrive at this aim, each holding to a different idea of justice [32].

The extent to which individual participants’ values – and the ethical perspectives they inform – accord with the principles underlying any given macroallocation exercise can shape that process, determining the content of the deliberation and the potential for arrival at common ground [26, 31]. Participants’ values can also influence how they perform their roles in ways that are independent of whether or not they subscribe to the values guiding the resource allocation decisions. For example, the values they hold about interpersonal engagement may influence how they interact to arrive at decisions.

That the expert generally promotes certain values and rarely functions in a purely technical role [26, 33, 34] brings into relief the privileged position of doctors and their limitations, and prompts our interest in understanding the ethical ramifications of their engagement in macroallocation. Since values figure conceptually prior to ethics, in that they help determine what is regarded as good or right [35], a description of the values held by doctors who engage in the normative evaluations inherent in distributive justice might form a useful stepping stone towards an ethics for this practice.

It has been claimed that the social conventions of policy processes militate against explicit and detailed exploration of values, and that unacknowledged differences in ethical perspectives amongst participants can cause conflict and failure to reach resolutions [14, 26, 36–38], a situation echoed in the empirical and theoretical literatures on priority setting where, although the plurality of values and ethical perspectives in the macroallocation process is acknowledged [9, 26, 37], little has been written on the values of the different actors, including doctors. It is common, instead, for assumptions to be made about the values and ethical frameworks doctors bring to resource allocation deliberations, notably that they have a deontological focus on addressing the needs of individual patients that contrasts with the utilitarian perspectives of most other categories of participant [26]. This divergence in frameworks is then used to account for difficulties in arriving at agreement on just allocations. The present study aims to identify the values of one group of participants: the doctors who play the role of technical expert in priority setting, so as to enable testing of some of the assumptions about their ethical perspectives.

Globally, under the influence of the physician charter of the ABIM Foundation [39], codes of medical ethics have begun to include as a professional commitment engagement in social and distributive justice activities [40–42]. The Australian Medical Association’s (AMA) encouragement of doctors to ‘use [their] knowledge and skills to assist those responsible for allocating healthcare resources, advocating for their transparent and equitable allocation’ is a typical example [43]. This trend has prompted intense debate about the ethics of doctors’ involvement in socially engaged actions [24, 40, 41, 44]; however, neither macroallocation as a setting for this commitment nor the details of its actualisation have been explored from an ethical standpoint. A central problem in the codes of ethics is the vagueness of the activities they embrace and of the descriptions of ethically relevant concepts, especially at the practical and social levels. For this reason, we believe that identification of the values of participants in macroallocation is a necessary foundation for normative work towards bridging the gap between medical professional ethics and the practice of socially engaged actions.

In order to obtain a rich picture of doctors’ understanding of the social process of policy and to bring their intuitions to bear on our ethical analysis [45, 46] we undertook a qualitative interview study of doctors who act as technical experts in macroallocation. Our enquiry elicited reflection on the roles doctors play in policy and their practical experience of those roles. Using the empirical bioethics methodology grounded moral analysis (GMA) [47], we considered doctors’ conceptual understandings alongside ethical theory in order to arrive at an understanding of ethics in macroallocation policy work. Our focus was on doctors who elected to engage in policy as individuals, rather than as representatives of interest groups, and who were focused on the resourcing of services rather than on the rights of medical practitioners or broad professional reform.

Our principal goals for this part of the project were: first, to develop an empirical account of macroallocation practice focusing on the values and ethical commitments of doctors who engage in it as technical experts; and second, by exploring with participants the normative implications of this information, to attempt to theorise an empirically grounded ethics for doctors’ role in macroallocation.

## Methods

### Methodology

This analysis is part of a larger qualitative interview study examining the ethical issues entailed in doctors’ participation as technical experts in government processes concerned with the allocation of resources to health care. To answer our research questions we selected sampling, data-collection, and analysis strategies that were based on Dunn et al.’s [47] GMA, an iterative empirical bioethics methodology founded in grounded theory (GT) [48] that employs contemporaneous interchange between the ethical and empirical to support normative claims that are grounded in practice.

### Participants and sampling

We recruited 20 doctors, each of whom had participated in macroallocation in the Australian health system. Ten doctors responded to an invitation issued on our behalf by the NSW Ministry of Health’s Agency for Clinical Innovation, which coordinates clinician networks that advise on aspects of health policy in NSW. Of these, one withdrew before an interview had been scheduled. The remaining 9 were interviewed, together with a doctor recruited by means of passive snowballing. In order to enable exploration of the categories emerging from the analysis we then undertook theoretical sampling from amongst members of our professional networks [49]. We issued 18 invitations in this stage, which yielded 12 affirmative responses. Out of these, 10 interviews eventuated, while for the remaining 2, theoretical saturation had been reached and data collection had ceased before interview arrangements could be finalised.

Each member of our sample had been involved as a technical expert in multiple macroallocation processes, acting concurrently or serially in policy at the institutional, local, state, national and, in some instances, international levels. All had advised or were currently advising policy makers in government macroallocation processes involving prioritisation of competing bids for health care funding. Their activities included: committee membership, meetings with bureaucrats and political decision-makers, lobbying, independent development of processes aimed at advancing debate on healthcare priorities, and preparation of submissions and correspondence. None were involved in championing the rights of medical practitioners, or in broad professional reform. Participants’ characteristics and their policy activities are set out in Table 1.

**Table 1:** Participant characteristics and policy activities

Sex (number of participants)	
Female (5)	
Male (15)	
Age Range (number of participants)	
≤ 45 (3)	
46–55 (4)	
56–64 (10)	
65–80 (3)	
Average age: 58	
Country of undergraduate training (number of participants)	
Australia (16)	
Europe (3)	
Asia (1)	
Clinical Specialty^a,b^ (number of participants)	
Paediatrics (3)	
Endocrinology (1)	
Plastic Surgery (1)	
Rehabilitation (3)	
General Practice Academic (2)	
Rheumatology (1)	
Clinical Pharmacology (1)	
Gastroenterology (2)	
Intensive Care Medicine (2)	
Neurology (2)	
Cardiology (1)	
Radiation Oncology (1)	
Policy engagement level and issue (number of participants)	
Multiple national governments or international bodies on public health programs (2)	
Australian government funding of healthcare research priorities (2)	
Australian government funding of high cost health care interventions (3)	
Australian government funding for priority healthcare programs (4)	
Australian government funding of participant’s specialty (2)	
NSW government funding of participant’s specialty (13)	
Local health administration funding of participant’s specialty (11)	
University priorities for health research and education program funding (4)	

^a^For participants with multiple specialist qualifications, the specialty listed is the one on which the majority of their policy work was focussed

^b^Participants’ subspecialties are withheld in order to preserve confidentiality

All of our participants were or had been (in the case of those who had retired) employed, mostly on a full-time basis, in the public sector. Age, sex, and country of training were distributed in the sample in accordance with their representation in the Australian specialist medical workforce [50, 51].

### Data-collection

The first author, who has experience as a participant in macroallocation, conducted the interviews, which lasted an average of 64 min, with a range of 30 to 90 min. Our semi-structured interview format was designed to draw out participants’ experiences and prompt ethical reflection. Our interview schedule was structured to explore broad topics initially, and became more focussed on participants’ moral intuitions as the interview progressed. We revised the interview schedule on two occasions, in response to our early normative and conceptual analysis, to enable the testing of emergent theories [47, 48]. In order to honour our commitment to GT-guided open-ended interviewing, we elicited later participants’ responses to ethical perspectives emerging from the data indirectly, rather than engaging them in a formal reasoning process or in explicitly validating a particular account; we elected not to make ethical theories explicitly visible to participants [47].

### Analysis

From the commencement of the interviews, we progressively undertook inductive and abductive data analysis, which involved preparing detailed codes, writing comprehensive memos, exploring relationships between codes, and combining codes to create analytic categories [48]. The analysis was conducted by the first author, who discussed emergent findings with the other authors, and drew reflexively on her own experiences in order to understand and take account of their role in informing the interpretation of the data [49, 52–55]. Since participants’ reflections on ethical matters were frequently implicit we acknowledge our interpretative role in constructing this account [56].

In our initial inductive analysis, we found that our participants shared strong ethical insights that tapped into themes of virtue ethics, particularly justice, practical wisdom, and moderation, to which we responded by developing our interview schedule in the direction of virtue ethics, in accordance with GMA methodology [47] and Salloch et al.’s advice [57] that ethical theories in empirical bioethics should be chosen on the basis of their appropriateness to the issue at stake and congruity with the moral deliberations of participants. We used the steps set out in GMA to validate our emerging ethical insights with participants [47]. The effect of this interplay between data and analysis was to recreate the ‘phenomenologically informed hermeneutic approach to ethics’ described by Rehmann-Sutter et al. [58], in which the authority of normative claims comes from the researcher’s ‘reconstruction of the ethical argument they have assembled during the collection of data’ [58].

In the course of our analytic and normative deliberations, we observed that the ethical themes in our data resonated strongly with Paul Ricoeur’s ‘little ethics’—a variant of virtue ethics that embraces self-esteem, solicitude, and participative justice [59].

### Ethics

The University of Sydney Human Research Ethics Committee gave approval for this study. Each participant gave consent in writing. All were assured that we would maintain confidentiality, and that they were free to withdraw from the study at any stage. We de-identified all data, and assigned alphanumeric codes for analysis and storage.

## Results

Participants’ accounts of macroallocation practice were rich in concepts of virtue, with justice, practical wisdom, moderation, and the virtues of self-efficacy [60] strongly represented. Participants also valued participation in the institution of macroallocation and had confidence in its power to effect just decisions. In addition, they gave their views on the ethically desirable and undesirable practices in which doctors might engage whilst performing the expert role, and offered recommendations for changing practice so that it more closely approached the ideal. Participants’ ideas of virtue in macroallocation practice were strongly aligned with the conception of virtue ethics set out by Paul Ricoeur in his ‘little ethics’. Ricoeur adds to the Aristotelian concept of the virtuous life a recognition of the generative possibilities of institutions [61].

In this section we describe the ethical features of our data; we relate them to the concepts in Ricoeur’s ethics in the following section.

**Fig. 1:**
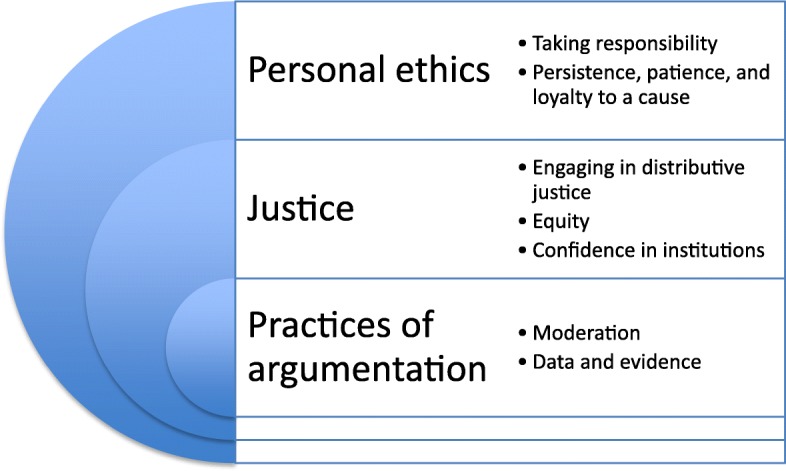
The values of doctors who engage in macroallocation work, as defined by participants

The doctors in our sample held values in common across three domains –personal ethics, justice, and practices of argumentation. These values are set out in Fig. 1 below and described in the text that follows.

### Personal ethics

#### Taking responsibility

Our participants believed that all doctors holding positions in public institutions had a duty to take up societally, educationally, or professionally focused roles in addition to their clinical work. It was a rare ‘brilliant clinician’ who deserved to be excused from this obligation. Most participants considered the responsibility to undertake macroallocation work to derive from their employment at the taxpayer’s expense in the public sector. For some it was a manifestation of gratitude for the privilege of working in a prestigious institution.

P8: This institution has been pretty good in allowing us to run around and do these things. I mean, that’s important.

Many of our participants considered complaining without attempting to address conditions in the health service to be unconscionable and viewed colleagues who did this as freeloaders, although some considered a structural feature of the Australian health system that makes it financially difficult for doctors in private practice to devote time to macroallocation to be a mitigating factor.

P15: I guess, it’s also the sense of being able to shape things and really having the bigger picture view of wanting to make sure – I guess you can’t complain about decisions and choices and policies if you’re not in there shaping them. So if you see things you don’t agree with, you’ve got to be in there doing it. So it’s that feeling of that responsibility.

Disapprobation of those who pursued this route for self-serving reasons, for example, as a means of building an empire or developing a career, was strongly represented in our data. Melioristic motives were offered by our participants for their own participation, and expected of others: for those whose focus was the local level, a desire to provide ‘the best service possible’ and reduce stresses on healthcare staff, and for those whose focus was state-wide, national or international, a desire to ‘make a difference’ and to ‘be of service’.

A small number of participants, even as they upheld authenticity and meliorism, admitted that they were unsure of their own motives.

P16: And for me, it’s a very uneasy feeling obviously, and I think it comes to the point where I then hope that what I achieve to advocate is worth more than what my ulterior motive was.

#### Persistence, patience, and loyalty to a cause

In our data the virtues of self-efficacy were highly valued: persistence, patience, and resilience were the qualities universally drawn on in macroallocation work. Persistence was particularly prominent across our data: our participants commonly evoked an image of themselves as ‘a dog with a bone’. We found that our participants set long-term goals and accepted deferred gratification. They endured failure and partial success sanguinely, but as an interim step towards achieving their objective, rather than the last word. Many spoke of trying different avenues when the first attempt failed, or repeatedly proffering the same opinion in anticipation of it finally hitting the target.

P1: I think you need to be able to justify what you need [in order] to be able to justify what you want, but then there are ways to get what you need. And maybe only half of what you need and that’s fine, or just, you know, take another three years to get the other half, that’s fine.

Our participants’ records bore out their claims: they remained committed to the issues and processes that had attracted their attention early in their careers, in some instances engaging with a particular process for decades. Although they had faced a wide range of negative experiences, including betrayal, disrespect, sexism, the consequences of being overcommitted, and high-profile public repudiation of their advice, they found it hard to think of circumstances in which they would give up.

### Justice

#### Engaging in distributive justice

A significant number of the doctors in our sample characterised themselves as protectors of the national purse. They were concerned about transparency, accountability, and waste in the health sector. For these doctors, engaging with practical matters of distributive justice was a natural response to those concerns. They considered that participation in policy making about resource allocation was a legitimate role for them, and for doctors generally, to play.

P20: You’re making decisions about allocation of the health care dollar in a cost effective and equitable way - so that is, in a way, you’re advocating for patients but you’re also advocating nationally for the country - so it’s not so much advocacy, but being at the pointy end of the way health care dollars are being allocated.

They believed in their own capacity to act in society’s interests and were confident that, without doctors’ experience and perspectives, decision-making would be impossible.

P6: So I guess the things – so I think that the system needs societal good, all right? And we probably have enough ego to believe that we can do – we can prescribe what that looks like.

The doctors in our sample declared no difficulty in separating this role from their obligations to individual patients; they spoke often about the different ‘hats’ they wore for different purposes.

P14: I think it has to be someone who’s interested in societal health and being an advocate for society because you’ve got to switch hats; if you’re a clinician and you’re treating a patient then clearly your role is to advocate for that patient and they would quite reasonably not like it if you took a societal view [*laugh*], [you] try and get the best you can for that patient, but if you’re making decisions you’ve got to put – take that hat off and put a different hat on.

Even as they engaged with the problem of apportioning healthcare resources, some of our participants shared an insight that decisions on resource distributions in health care were essentially arbitrary and that investing in non-health programs might be equally, if not more, justifiable in terms of benefit to society.

#### Equity

Of the 20 participants, 11 had entered an emerging specialty or subspecialty, or had identified and developed their practices to focus on a previously unrecognised issue; almost all of the remainder occupied niches in evolving areas of their specialties. Most participants expressed a view that the disease entity in which they were interested was considered unattractive, or that their patient groups were neglected or stigmatised. Some were interested in securing access to services for those who were socially marginalised or geographically disadvantaged. A number of our participants considered themselves to be the ‘champions’ of such groups and issues, while at the same time regretting the need for champions. It was common for participants to express wry resentment about how easy it was for peers seeking resources on behalf of ‘sexier’ illnesses or patient groups to gain advantage.

P14: Their catchcry for that was ‘this is an evidence based decision making process, not an advocacy based process’, because most health is about my disease is bigger than your disease and if you do a cost of illness study, everyone’s disease is the biggest and all of that, but my prime motivation was actually for advocacy for [the patients of my specialty] because they are the poor cousin.

#### Confidence in institutions

In our participants’ accounts, macroallocation was experienced as a rewarding practice. They enjoyed being valued by bureaucratic and ministerial decision-makers, and considered that long-term commitment to macroallocation eventually yielded benefits, built reputations, and created further opportunities. They were motivated by their confidence in the policy and political processes available for determining resource allocation and valued engaging with them as a worthwhile activity from which they derived both results and feelings of belonging and worth. Almost all of our participants saw the bureaucrats with whom they engaged on policy as well-meaning, dedicated people trying to do the right thing. They believed bureaucrats largely to be responsive to the evidence provided by doctors, and willing, capable interpreters of doctors’ perspectives to political decision-makers. They were optimistic about their chances of success in the deliberative process.

P6: Lots of [doctors] I guess denigrate, in my view quite unfairly, the hierarchy of the bureaucracy, and what bureaucracy can do for them. I guess it's fair that I don't see bureaucracy as intimidating or frightening or - they're there to be - like everyone they're there to do their job and they are open to persuasion like everyone else is. And open to be guided to make a better decision.

Some, however, characterised their interaction with bureaucrats as ‘playing the game’. Whether cynical or supportive, our participants all worked hard to assemble the ‘case’ that they believed would satisfy the bureaucrats’ need for information to ‘take up the line’ to decision-makers. A small minority claimed to distrust authority or dislike bureaucrats of all stripes, although even these individuals had forged successful relationships with bureaucrats who supported them.

We found that the doctors in our sample understood the constraints on their influence in the deliberative processes to which they belonged, and believed their advice was for others to adjudicate upon.

P5: Even if it’s not an outcome I agree with, if the process is fair then I’m happy with that.

### Practices of argumentation

#### Moderation

Our participants valued balance and moderation in their formal transactions in macroallocation. A number said they steered clear of melodrama or ‘overhyping’ in order to avoid alienating bureaucrats or causing ‘the bullshit meter to go up’ (P6). P20’s description of his approach was typical:

P20: You have to persuade governments, not shout at them or bully them.

Universally in our sample, doctors who flouted the rules or gained advantage through being ‘squeaky-wheels’ attracted disapproval. A special level of condemnation was reserved for those who went to the media, or who leap-frogged the chain of command, especially if they invoked the intervention of a politician. P8’s characterisation of such actors as ‘Visigoths’, who were looking after their own agendas, exemplified our sample’s view. Some of our participants described how groups self-policed to bring colleagues into line or to weed out those who could not operate in accordance with the group’s culture.

P4: [This committee is] here to advocate for society as it were and people work that out pretty smartly and, if they don't, they don't stay or they get booted off.

A small number of our participants, although endorsing moderate tactics, recounted incidents where they had thumped tables, engaged in ‘stand-up rows’, importuned government ministers, and bypassed the chain of command. Two claimed to understand the frustration of actors who did not play by the rules, and had questioned their own tactics, on the basis that they were ‘too nice’ to be successful.

#### Data and evidence

We found that our participants were committed to the use of numerical data, evidence based medicine (EBM), and cost effectiveness analysis in the policy process, and were confident in the capacity of these techniques to compel a response. Observations such as P1’s, that ‘any doctor with a cause must master the data’, were common. Most participants went to considerable lengths to gather and present convincing data, including acquiring skills in healthcare and financial data analysis, establishing purpose built databases, and operating complex research programs designed to allow quantification of patient and service needs. They saw themselves as holding back the tide of irrationality, disparaging colleagues who did not share their regard for data as the foundation for argument, especially if they dismissed available data or countered evidence with anecdote. Paradoxically – since it did not disrupt their commitment to using data – many participants reported dismay at the frequency with which someone with no data but a good story swayed opinion, and some noted that deploying clinical vignettes assisted them in argumentation. A number reflected on the gap between the desire for evidence-based decision-making and its execution.

## Discussion

In addition to the solidaristic values that are commonly held to underpin macroallocation, our participants shared epistemological values and concepts of virtue that informed the way they practiced macroallocation. We found that the doctors in our sample held values in common across the domains of personal ethics (‘taking responsibility’ and ‘persistence, patience, and loyalty to a cause’); justice (‘engaging in distributive justice’, ‘equity’, and ‘confidence in institutions’); and practices of argumentation (‘moderation’ and ‘data and evidence’).

Through our use of GMA, which involved iterative juxtaposition of data and theory, and engagement of participants in exploring ethical insights emerging from the data, we were able to establish that our participants’ conceptualisations of the good in macroallocation practice resided in a seam of virtue ethics that was congruent with Paul Ricoeur’s ‘little ethics’. In this discussion we reflect on the explanatory and normative implications of this finding and integrate the empirical and theoretical ideas we developed in our analysis.

Ricoeur’s ethics – encapsulated in the ethical aim of ‘the “good life”, with and for others, in just institutions’ – is based in his work on hermeneutics and narrative identity. It was initially set out in his book *Oneself as Another* [59] and elaborated in his later writings, including *Reflections on The Just* [62]. It embraces a ‘social ontology of solicitude, care, promising, and accountability’, and recognises that the life of each ‘other’ is as significant as one’s own [63]. A form of virtue ethics, it is founded on the claim that human life has an ethical aim: self-esteem, expressed as the need to approve one’s own existence and be approved of by others and, as such, it entails ‘personal pride about being oneself’ [62].

Ricoeur’s idea of ‘the good life’ draws on the Aristotelian concept of *eudaimonia*, or flourishing, and his notion of ‘living with and for others’ reflects the subject’s relationship of reciprocity and mutuality with other people [59]. What distinguishes his ethics from other virtue ethics systems is its treatment of ‘just institutions’ as a fundamental component of a good life [62]. For Ricoeur, the concept of ‘the just’, which is assigned the meaning ‘equitable’ [64], pervades all human actions [62].

In Ricoeur’s ethics just institutions mediate equity, expand the ethical focus beyond single, identifiable others, and cultivate acts of the human spirit [65]. Deliberation in the just institution is founded on the exercise of practical wisdom – the Aristotelian concept of *phronesis* [62] – and supported by obligations to make the best possible argument [62] and engage respectfully with the convictions of others [64]. Respect and mutuality in deliberation are evoked by the act of translation from the language of one’s own ‘culture’ into that of the other; Ricoeur calls this phenomenon ‘linguistic hospitality’ [62]. Because we had integrated virtue ethics into our data collection and preliminary analysis we were able to apply Ricoeur’s version of virtue ethics into our normative considerations in response to the strength with which these features were expressed in our data.

Neither Ricoeur’s ‘little ethics’ nor his work on medical ethics has been widely referenced in the biomedical literature [66, 67]. Although his detailed work on the medical role is confined to clinical care and, to a lesser extent, research [62], he identifies the ethical challenge of doctors’ involvement in matters of public health and public expense [62]. On this basis, and because of the centrality of distributive justice in his thinking, we believe his ethics may illuminate the medical role in macroallocation.

The concordance between our empirical data and the three levels of Ricoeur’s ethics lends weight to the idea that it might serve better than either virtue ethics or deontology as an ethical framework for doctors undertaking macroallocation work, and as the foundation for actualising the medical ethics codes’ promotion of engagement in resource allocation.

### Personal ethics

#### Taking responsibility

Our participants valued performing the medical role in its widest sense and took responsibility for addressing systemic problems they encountered in their clinical roles; they expressed a strong antipathy towards impure motives. Their rationales for taking part in macroallocation were consistent with theoretical positions on the relative importance of personal ideals and extraprofessional ethical codes vis a vis the deontological medical codes in shaping doctors’ professional behaviours and choices [68–70]. Their accounts of their motivations suggest that macroallocation practice is located within concepts of virtue, and within an idea of the good life that includes professional flourishing and melioristic, socially responsive, and solidaristic actions.

It was these features of the data that evoked the first level of Ricoeur’s ‘little ethics’. Ricoeur’s idea of ‘the good life’ draws on the Aristotelian concept of *eudaimonia*, or flourishing. The subject’s aim of a ‘good life’, which Ricoeur also terms an ‘accomplished life’ [62], includes professional excellence [62] and virtuous actions, which, when interpreted (favorably) by the subject, become self-esteem, or ‘personal pride about being oneself’, at the ethical level [59, 62]. For Ricoeur, ethics has primacy over morality, and solicitude primacy over duty [59].

Whilst there is much in this level of Ricoeur’s ethics that applies equally to clinical practice and policy work – responding to the suffering of others, being someone others can count on, taking responsibility, mutuality and caring – it is in joining this level with the notions of ‘working with and for others in just institutions’ that the possibilities of wider projects dealing with the public’s health and the public expense emerge. Ricoeur observes that the term ‘responsibility’ entails the notion of ‘responding’, and means both ‘counting on’ and ‘being accountable for’ [59]. Responding to perceived deficiencies in health care on behalf of others in aggregate – both groups of patients and society at large – thus fosters the testimony or attestation that is the culmination of Ricoeur’s ethics [63], allowing recognition of oneself as the one who is called to respond to the suffering of the other [71] and enabling the modest self-esteem that the ethical aim fosters.

The close fit between the first level of Ricoeur’s ethics and our data on policy-active doctors’ aspirations and perceptions suggests that this ethical system has value as an explanatory and normative matrix for doctors’ approach to the expert role. Its normative relevance is conferred by the standards of ethical reasoning it contains, which have the potential to guide doctors towards right action [23, 72] when evaluating the place of macroallocation practice in the good life and reflecting on how it might be conducted authentically.

#### Persistence, patience and loyalty to a cause

The intensity of our participants’ valuations of persistence, patience, and loyalty to their chosen causes despite frequent and severe setbacks suggests that macroallocation plays essential anchoring and projecting roles in their lives, and invokes Ricoeur’s notion of self-constancy, whose role in Ricoeur’s account of human flourishing may enable us to extend our theorisation of this phenomenon beyond the bounds set by traditional virtue ethics approaches.

Ricoeur’s concept of ‘selfhood’ is the *ipse –* or narrative – identity, which can change ‘within the cohesion of one lifetime’ [73]. The *ipse* identity, constituted as self-constancy, is guaranteed by promising: keeping one’s word to others offers ‘a faculty for mastering the future as if it were the present’ [74]. Promising entails being ‘pushed into future action by a projected self’, who is a person who can be counted on to keep their word [75], thus fostering the self-esteem that inheres in the ethical life.

We hypothesise that, since future-building, except in the most abstract sense, is not feature of clinical practice, especially under modern care models, macroallocation played a vital role in enabling the doctors in our study to flourish by acting as a temporal projection of their influence and intent, enabling them to deliver on their promises to the ‘others’ that are the objects of their interest and, ultimately, actualise self-constancy.

### Justice

#### Engaging in distributive justice, and equity

Our participants made long-term commitments to policy work in macroallocation, a field occupied with distributive justice. They strongly expressed commitments to cost effectiveness, clinical effectiveness, justice, equity and solidarity, which align with the content values typically associated with macroallocation [31]. To our knowledge, this correspondence, although unsurprising, has not been reported previously in the empirical literature on substantive values in priority setting.

This finding resonated strongly with Ricoeur’s ethics, in which apportioning society’s resources is the aim of acting together in institutions and contributes to the actualisation of the ethics of a good life [65]. The status of equity at the heart of Ricoeur’s ethics, as the element that links the subject to others and expresses the communitarian aim, resonates with our participants’ strongly voiced solidaristic values and appetite for engaging in distributive justice.

That justice is the objective of health policy deliberations is widely understood [10, 76–79]. Although the doctors in this study valued distributive justice, they were unfamiliar with formal substantive principles for guiding resource allocation, and lacked consciousness of the irreconcilable conflicts that exist between them [80, 81].

Conflicting conceptualisations of justice and disagreement on principles for prioritising have led to a focus in macroallocation on procedural justice approaches [81–85]. Neither the specific process values proposed by Daniels, Sabin [86] in their influential ‘accountability for reasonableness’ framework– relevance, publicity or transparency, the possibility of appeals and revision, and regulative/enforcement mechanisms – nor those in Clark & Weale’s [31] framework – transparency, accountability, and citizen and patient participation – were present in our data.

A possible explanation for these two findings is that doctors are insulated from reflecting and acting on substantive and process criteria by virtue of occupying the role of expert rather than process designer, and of the general tendency for values to be glossed over in priority setting [14, 26, 36–38]. We also found amongst our participants little insight into the extent or consequences of the arguably undesirable, and certainly inequitable [20–22, 87] privilege the doctor’s voice is accorded in macroallocation.

Our finding of doctors’ distance, notwithstanding their valuation of justice, from key normative features of contemporary macroallocation is concerning, and suggests a need for macroallocation projects to develop shared understandings amongst participants of distributive justice ideals and ethical systems.

#### Confidence in institutions

Our participants experienced macroallocation as personally and professionally rewarding, and were confident in its potential to deliver just allocations. In this respect, they embodied Ricoeur’s recognition of the generative power of institutions. In their views on bureaucrats and politicians, however, they departed from the standard of solidarity and solicitude required by Ricoeur’s ethics. Bureaucrats were conceptualised by the doctors in our sample as malleable resources: nurtured within the bonds of shared interests and given the right information, they would serve doctors’ ends. The notion of the just institution in Ricoeur’s ethics demands moral and epistemological humility, and recognition of the equal worth, moral competency and convictions of the other [59, 88], to which the blurred sense of bureaucrats as independent agents found in our data is antithetical.

The ‘little ethics’ gives ethical shape to doctors’ valuation of justice, but in doing so, exposes areas where their aspirations are not met in practice. In Ricoeur’s ethics, ‘just institutions’ support both the ethical aim of the ‘self’, who exercises a ‘sense of justice’ within them, and the care of the ‘other’ who receives a fair share as a result [89]. Whilst the notion of distributive justice and the potential of institutions to dispense it appeal and give sustenance to doctors who engage in macroallocation, our findings suggest that structural factors in policy-making prohibit their grasping at a deep level the aim of justice in this context; these factors may skew the actualisation of the third level of Ricoeur’s ethics towards those receiving justice as a ‘share in’, at the expense of those receiving justice as a ‘share of’. Were it to be employed as a guide to ethical action in macroallocation, Ricoeur’s ethics, which gives due weight to each of these dimensions, would mitigate this possibility by bringing into sharper focus the ultimate ethical objective of justice.

### Practices of argumentation

Our participants valued practices of argumentation that mitigated the threats to justice arising from the structural factors we have described. These values – moderation and respect for data and evidence – demonstrated that our participants shared a preference for respectful deliberation and an intuitive grasp of *phronesis*, the Aristotelian concept of practical wisdom that defines the third level of Ricoeur’s ethics [59].

#### Moderation

Our participants strongly maintained that doctors participating in macroallocation should have the propensity for moderation. Although aware of, and tempted by, more aggressive ways of performing the expert role, our participants’ claims that they remained largely temperate suggests that they recognised moderation as essential to performing the role ethically. Their weighing of the merit of each extreme may be a reflection of the place of virtue in their ethical reasoning systems.

Moderation is in harmony with the Aristotelian concepts of virtue contained in first level of Ricoeur’s ethics and with the features that most strongly support arriving at just allocations: deliberative justice as a project of mutuality [90], recognition and respect for the convictions of the other and, ultimately, willingness to compromise rather than attempt to win by force [88].

The theme of compromise was also strongly represented in our data. Ricoeur’s ethics, in focusing on the interaction between individuals rather than on personal integrity as a response to plurality in values and voices, recognises compromise as a moral duty [88] and supports assertions in the theoretical literature on policy-making that respectful deliberation culminating in agreement on what are ‘good’ or ‘right’ actions are the normative foundation for dealing with plurality [91, 92]. To Ricoeur, determination to act on principle in this context is unethical because it fails to recognise and respect the worth of the other [88]. In our data, it is what distinguishes the ‘Visigoth’ from the ethical practitioner.

According to Yeager & Herman’s [88] reading of Ricoeur, moderate deliberation, by exposing convictions to proper evaluation, not only encourages equitable distributions but also offers to the subject the chance to understand herself as one amongst others and to experience a new dimension of personhood through the act of modifying her convictions. We hypothesise that macroallocation has a valuable function for doctors whose professional lives do not otherwise offer many opportunities for self-scrutiny and ethical development.

Ricoeur’s ethics has been criticised for being unreasonably optimistic about the extent to which mutuality prevails in deliberation, participants’ willingness to engage in ‘linguistic hospitality’, and the self-scrutiny that is required to adjust convictions and allow compromise [90]. Indeed, Ricoeur recognises the courageousness of his vision [62]. Our data showing that individuals are capable, despite inherent and external obstacles, of selecting and following an ethical course, suggest that these objections do not diminish the value of Ricoeur’s ethics – with its sensitivity to practices of mutuality and respectful debate – as a normative guide for those engaging in macroallocation.

#### Data and evidence

Performing the expert role typically involves repackaging clinical experience into data that is expressed in the language of the bureaucrat [26, 93] and directed towards satisfying the requirements of the neopositivistic policy-making models currently favoured in health policy-making [91, 94–96]. This was the experience of our participants, who held profound epistemic commitments to data and evidence based medicine (EBM), and strong ethical commitments to obtaining the skills required to deploy them effectively in argumentation.

Ricoeur recognises that deliberators in distributive justice often assign different meanings to language, concepts and customs, necessitating translation between parties [90]. In the literature on health care policy making, it is commonly claimed that the direction of translation is from expert to bureaucrat [26, 93]. Ricoeur refers to the act of translation as ‘linguistic hospitality’ and considers it to be an ethical act of caring and mutual welcome [62]. Although our participants’ willing cooperation in this endeavour exemplifies linguistic hospitality, by virtue of the unilaterality they describe, it lacks the mutual generosity, charity and respect for the others’ convictions that Ricoeur considers essential if debate is to foster the social cooperation and flourishing that is called for by the ethical intention [90]. In the context of macroallocation, doctors’ preoccupation with adopting bureaucrats’ language could further distance the already under-represented patient from deliberations, and efface representations of suffering from them. Ricoeur’s focus on mutuality would offset this risk were linguistic hospitality employed in an ethical guide for doctors’ macroallocation work, by bringing into relief the reciprocal responsibilities of other actors. This feature of Ricoeur’s ethics suggests its potential as an ethical guide to deliberative interactions for macroallocation participants more broadly.

The relevance of Ricoeur’s accounts of *phronesis* and ethics in deliberation emerged from our data on practices of argumentation in priority setting, suggesting that Ricoeur’s ethics might have roles to play in the development of norms for doctors’ deliberative practice in macroallocation and in the rectification of discrepancies between what is valued and what is performed. The mutuality, linguistic hospitality, and respect for the convictions of others demanded by Ricoeur’s ethics suggests that it might also have relevance as a guide to practice for the other actors in health care priority setting, especially in a future in which patient and community involvement in priority setting is routine [97–100].

## Limitations

We included doctors from a wide range of specialties, and our sample matched the profile of the specialist workforce in Australia [50, 51] for age, sex, and country of training. In addition, some participants had international experience in the role. We believe, therefore, that our participants’ experiences are are likely to be consistent with those of doctors who engage in macroallocation in formal processes in other socialised health systems. We do not report on distinctions between sub-groups, and so cannot say whether such distinctions might be significant. Our findings represent a single social perspective due to our focus on data only from doctors. Further empirical research into the ethical dimensions of doctors’ participation in macroallocation and into the experiences of other actors in priority setting would be needed in order to obtain a more comprehensive picture of the phenomenon.

## Conclusions

In this analysis, we explored priority setting as an ethical practice and investigated the social and moral meaning of doctors’ engagement in it. We described and explored the theorisation of values in macroallocation work, and brought together practical and theoretical domains not previously united for the purpose of moving towards an ethical framework for doctors’ role in macroallocation.

We found that the values of macroallocation practitioners covered domains of personal ethics (‘taking responsibility’ and ‘persistence, patience, and loyalty to a cause’); justice (‘engaging in distributive justice’, ‘equity’, and ‘confidence in institutions’); and practices of argumentation (‘moderation’ and ‘data and evidence’). To our knowledge, this information has not been previously derived empirically. The findings challenge the assumption that doctors in the priority setting milieu are motivated primarily by deontological considerations. The broad span of ethical commitments we have described offers novel insights into the social and ethical processes at play in this setting and opens up opportunities for understanding how doctors’ values might have procedural and substantive impacts on macroallocation.

Our use of the principles of GMA enabled Ricoeur’s ‘little ethics’ to emerge from our data as an ethical framework for medical work in priority setting that reflected doctors’ ideas of the good in macroallocation and their normative insights into how it might be performed ethically. The close fit between our data and the three levels of Ricoeur’s ethics suggests that this ethics system is a valid explanatory and normative model for doctors’ expert role in macroallocation. Practitioners of the role might find value in Ricoeur’s ethics as an aid to ethical reflection about their performance of the role, as a guide to ethical action, and as a foundation for resolving discrepancies between values and practice. Its embrace of mutuality and respect for the convictions of others suggests that the ‘little ethics’ might also have a role in harmonising deliberative practices amongst macroallocation actors generally.

## Abbreviations

EBM: evidence based medicine
GMA: grounded moral analysis
GT: grounded theory
